# Schwannoma of the vagus nerve, a rare middle mediastinal neurogenic tumor: case report

**DOI:** 10.1186/1749-8090-4-68

**Published:** 2009-11-26

**Authors:** Kyriakos St Rammos, Stylianos K Rammos, Christophoros N Foroulis, Thomas K Zaramboukas

**Affiliations:** 1 Department of Thoracic and Cardiovascular Surgery, Aristotle University Medical School, Thessaloniki, Greece; 2 Department of Neurosurgery, Medical College at Peoria, University of Illinois, Urbana-Champaign, USA; 3 Laboratory of Pathology, Aristotle University Medical School, Thessaloniki, Greece

## Abstract

Schwannoma originating from the vagus nerve within the mediastinum is a rare, usually benign tumor. A 44-year old male was presented with chest pain. Chest radiography, CT scan and MRI showed a well circumscribed mass, 5 × 4 cm located in the aortopulmonary window. The mass was found at surgery to be in close proximity with the aortic arch and the left pulmonary hilum, alongside the left vagus nerve. The encapsulated tumor was completely resected through a left thoracotomy incision and it was found to be a benign schwannoma in pathology. The patient is free of recurrence 6 years after surgery.

## Background

Neurogenic tumors represent approximately 20% of all adult and 25% of all pediatric primary mediastinal neoplasms. They are divided into nerve sheath, ganglion cell and paraganglionic cell neoplasms [1]. Neurogenic tumors are benign mediastinal tumors with rare exceptions [1-3]. Schwannomas or neurilemmomas originating from the vagus nerve are rare mediastinal tumors, accounting for 2% of all mediastinal neurogenic tumors, arising typically from the nerve sheath and extrinsically compressing the nerve fibers [1,4].

Scwannomas are lobulated, encapsulated spherical masses, different from neurofibromas in that matter. Men and women are equally affected in their third and fourth decades [1]. Usually, they are asymptomatic and benign, and very rarely malignant or multiple [2-5]. Shwannomas usually arise from a spinal nerve root, indeed they may arise from any other intrathoracic nerve [1,4]. Radiologically they are sharply demarcated with rare calcifications. CT contrast enhanced scan of the chest shows in accordance, a sharply demarcated mass with low densities and mild enrichment, rarely with calcifications and no fat. On MRI the schwannomas have low - to intermediate signal intensity on T1-weighted images and may have intermediate - to high - signal intensity on T_2_-weighted sequences [6,7].

## Case Presentation

**Figure 1:**
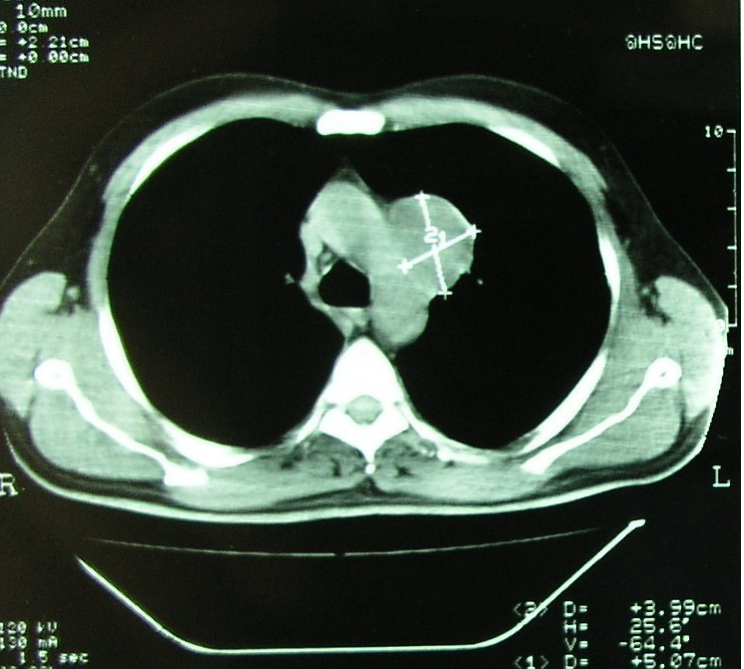
**CT contrast enhanced scan of the chest showing the smooth and clear margins of the mass and its location inthe aortopulmonary window**.

**Figure 2:**
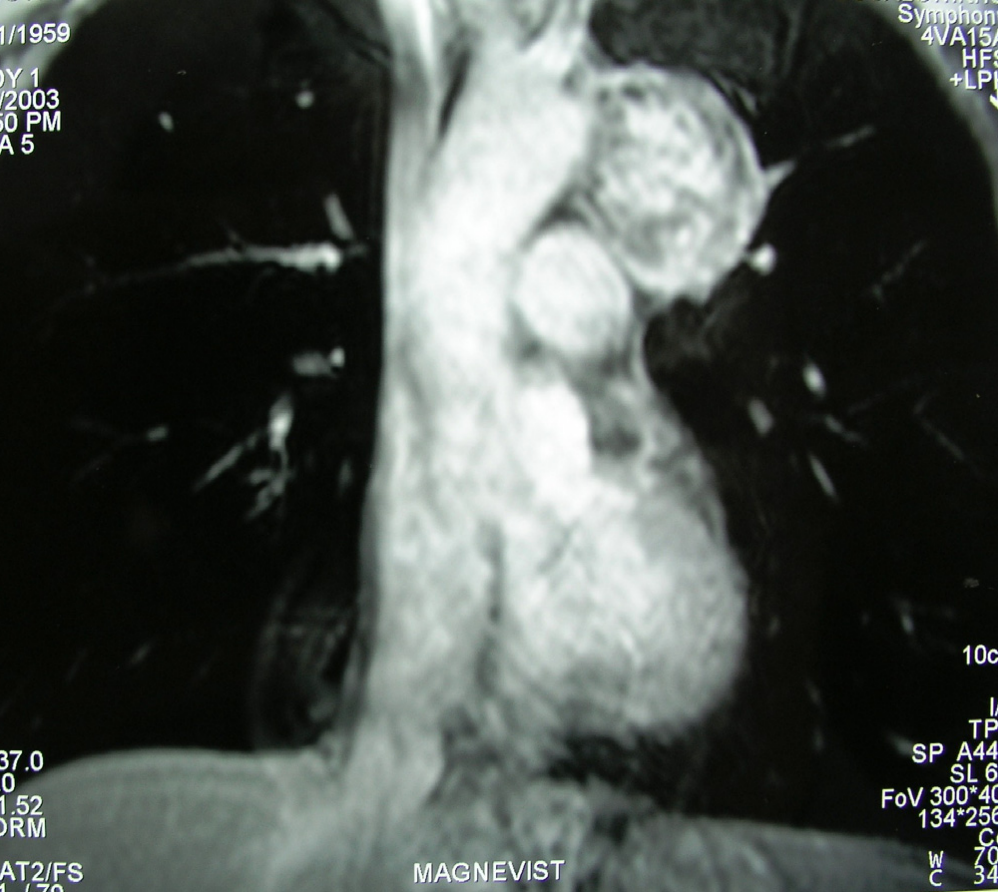
**MRI of the chest showing an encapsulated mass in theaortopulmonary window, suggesting a benign lesion**.

A 44-years old male presented with a sense of heaviness and pain in the left anterior chest wall. Preoperative chest radiography showed a sharply demarcated extrapulmonary mass withour calcifications in the middle mediastinal compartment, between aortic arch and left hilum. Contrast-enhanced CT scan of the chest showed a 5.1 × 4 cm mass in the aortopulmonary window, with smooth and clear margins, low densities (HU: 9-15) and light enrichment after administration of contrast material (HU: 22-37), without calcifications and fat (Figure 1). On MRI, the tumor showed the characteristics as previously described (Figure 2).

**Figure 3:**
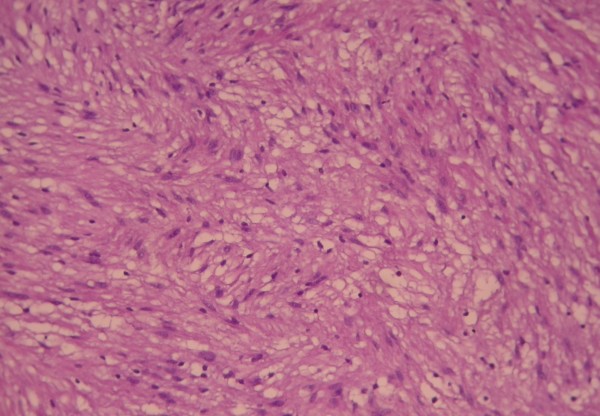
**Spindle cells which are arranged in fasicles in a loosestroma (HEx200)**.

**Figure 4:**
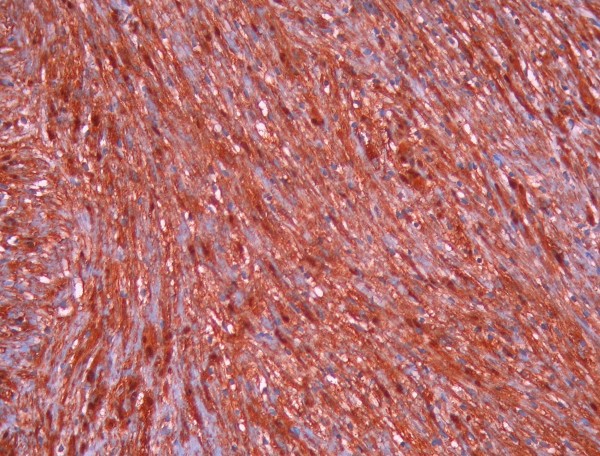
**Strong positivity for S-100 protein of the tumor cells (×200) suggesting schwannoma**.

He underwent a left anterolateral thoracotomy in the 4^th ^intercostal space preserving the latissimus dorsi instead of VATS procedure because of the proximity of the tumor to the aortic arch and the left main pulmonary artery. Complete resection, as is the case for benign schwannomas, was performed of this 5 × 4 cm tumor, located in close proximity to the vascular structures of the aortopulmonary window, alongside the left vagus nerve which was left intact. The tumor was removed with its entire capsule and was found to be benign at histology, because there were no atypia, mitoses, increased cellularity and necrosis. (Figure 3 and Figure 4)

The patient had no postoperative complications and he is free of recurrence 73 months later.

## Comment and Conclusion

Benign schwannomas of the vagus nerve are very rare middle mediastinal neurogenic tumors of nerve sheath origin [1,2]. Schwannomas of the vagus nerve occur usually on the left hemithorax while they may reach huge dimensions in rare instances [1,2]. Thoracotomy instead of VATS was preferred in the presented case because of the location of the tumor in the middle mediastinal compartment, close to the aortic arch and the hilum of the left lung. VATS resection is an alternative option for resection of mediastinal neurogenic tumors however the location of the tumor in the middle mediastinum and especially in the aortopulmonary window may necessitate thoracotomy for safe isolation of the tumor from the vital mediastinal structures and further, in order to avoid damage to the recurrent nerve during dissection of the tumor within the aortopulmonary window [8-10].

The tumor was confirmed to be a schwannoma because microscopically it showed spindle cells in fascicles in a loose stroma. Strong positivity of the tumor cells for S-100 protein confirmed the diagnosis of schwannoma [3-7]. Malignant schwannomas are rare and they are distinguished from benign schwannomas on microscopic examination because they show atypia, mitoses, pleomorphism and necrosis [3,4,11]. More than fifty percent of malignant schwannomas are found in patients with neurofibromatosis [1].

The long-term survival after complete resection of the extremely rare malignant scwhannomas of the vagus nerve seems to be satisfactory in sporadically reported cases [3,9].

## Consent

Written informed consent was obtained from the patient for publication of this case-report and any accompanying images. A copy of the written consent is available for review by the Editor-in-Chief of this journal.

## Competing interests

The authors declare that they have no competing interests.

## Authors' contributions

All authors have read and approved the final manuscript. KSR performed the operation, has been involved in drafting the manuscript and has given the final approval to publish the manuscript. SKR has been involved in critically revising the manuscript. CNF has made contribution to design of the manuscript and has been involved in drafting and critically revising the manuscript. TKZ did the histology and immunohistochemistry of the tumor and has been involved in drafting the manuscript
